# An Improved Approach of Mesh Segmentation to Extract Feature Regions

**DOI:** 10.1371/journal.pone.0139488

**Published:** 2015-10-05

**Authors:** Minghui Gu, Liming Duan, Maolin Wang, Yang Bai, Hui Shao, Haoyu Wang, Fenglin Liu

**Affiliations:** 1 College of Mechanical Engineering, Chongqing University, Chongqing, China; 2 Engineering Research Center of Industrial Computed Tomography Nondestructive Testing of the Education Ministry of China, Chongqing University, Chongqing, China; Chongqing University, CHINA

## Abstract

The objective of this paper is to extract concave and convex feature regions via segmenting surface mesh of a mechanical part whose surface geometry exhibits drastic variations and concave-convex features are equally important when modeling. Referring to the original approach based on the minima rule (MR) in cognitive science, we have created a revised minima rule (RMR) and presented an improved approach based on RMR in the paper. Using the logarithmic function in terms of the minimum curvatures that are normalized by the expectation and the standard deviation on the vertices of the mesh, we determined the solution formulas for the feature vertices according to RMR. Because only a small range of the threshold parameters was selected from in the determined formulas, an iterative process was implemented to realize the automatic selection of thresholds. Finally according to the obtained feature vertices, the feature edges and facets were obtained by growing neighbors. The improved approach overcomes the inherent inadequacies of the original approach for our objective in the paper, realizes full automation without setting parameters, and obtains better results compared with the latest conventional approaches. We demonstrated the feasibility and superiority of our approach by performing certain experimental comparisons.

## Introduction

In practice, mesh segmentation for region extraction as applied to a triangular surface mesh is a key prerequisite for subsequent major procedures (such as texture mapping, remeshing and quadrangulation)[1], therefore currently it has been a significant and popular research topic for decades in computer graphics. In surface graphics, feature regions are defined as those surface areas with relatively extreme geometry, i.e., the concave and convex regions in the surface model.

Regarding mechanical parts, for reason that their surface geometry exhibits dramatic variations and their concave and convex features are equally important during modeling, the targeted approach of mesh segmentation for feature region extraction should differ from those previous ones used on natural surface mesh models whose surface geometry exhibits slight, gradual variations and concave features are relatively important compared to convex features in cognitive science. Therefore, this paper presents an improved mesh segmentation approach based on an original approach used specially for natural surface mesh models in principle of cognitive science, to extract feature mesh regions that composed of concave and convex feature regions, from the surface mesh of mechanical parts.

According to the objectives, mesh segmentation is classified into two types: semantic segmentation and geometric segmentation [1]. Geometric segmentation relies on local surface properties to divide the surface into a few regions each of whom is composed of elements with certain desired geometry, whereas semantic segmentation is to divide the model into meaningful parts and does not need to consider local surface properties [2]. Extracting feature mesh regions in the paper is categorized as geometric segmentation in the sense that they have the same objective of obtaining regions with certain desired geometric property.

For details on geometric segmentation, readers are referred to the literature [3]. Here, we list a few most important related works. In [4,5,6,7], mesh segmentation was conducted by fitting analytic surfaces to the mesh for reconstruction of a CAD model. In [8,9], the objective function was constructed by creating a database, and the database was then applied to the targeted surface mesh for analysis to obtain the resulting segmentation. In [10], a Gaussian map was used to evenly distribute the Gaussian curvatures on the resulting charts to segment globally closed surface mesh model. In [11], fast approximate convex decomposition (FACD) was proposed and used to improve the quality of mesh segmentation by providing a strategy for evaluating potential cuts with the goal of reducing the relative concavity. In [12], an automatic mesh segmentation framework was presented and implemented through hierarchical spectral analysis and isoline-based boundary detection. Using the visual perception of human being, the approaches derived from cognitive science [13,14] were developed to extract concave feature regions from a natural surface mesh model. Moreover, mesh segmentation for extracting feature regions may also be interpreted as identifying the C^1^ discontinuities of a surface mesh. As one of the most representative algorithms in [15], an indicator used to identify C^1^ discontinuities as features was derived from the error in local fitting by a paraboloid, and the results showed that the indicator is insensitive to mesh dimension dispersion.

Among the above listed techniques of geometric segmentation, we attempt to find one or more solution(s) that are suitable to the objective of this paper to extracting feature regions. Feature regions of a surface model are always located where meaningful parts intersect; therefore, they often have complex geometry and are difficult to be expressed mathematically. Thus the approaches of fitting analytic surfaces in [4,5,6,7] are inappropriate for extracting feature regions. The learning approaches in [8,9] based on constructing an objective function are cumbersome and not mature in practice; furthermore, due to the uncertainty of the complex geometry of feature regions, it is difficult to realize the learning approaches to extract them. In [10], the use of Gaussian map to evenly distribute Gaussian curvatures obtains surface mesh regions as developable as possible, and thus its goal is different from this paper. The FACD approach in [11] was originally designed to improve the effect of segmentation, and its results were regions with similar geometrical property instead of feature regions. As for the approach in [12], it was the one which is more likely used for boundary detection. In summary, none of the above enumerated approaches of geometric segmentation is just suitable to the objective of the paper.

While the approaches of identifying the C^1^ discontinuities might be considered to partly correspond to our objective. As for the approach in [15] to identify the C^1^ discontinuities, for reason that its indicator is a fitting error which couldn’t directly determine a sharp geometry, therefore a few false C^1^ discontinuities might be identified and a few apparent features may not be identified suffering from somewhat complex cases. Meanwhile, the approaches derived from cognitive science in [13,14] were just to extract feature regions from surface mesh models, but they could only extract concave feature regions from natural surface models whose surface geometry gradually and smoothly varies and concave features are relatively important in visual recognition of human being. However, in this paper, the subject objects are mechanical parts to be modeled whose surface geometry strongly varies and where the convex features are as important as the concave features. Therefore, the results of the two cognitive approaches are not perfect and need to be supplemented.

Thus, to achieve appropriate results on extracting feature regions for modeling mechanical parts, a novel approach that represents an improvement over the previous approach in [13] is presented in the paper, and its indicator is the processed values of the minimum curvatures on vertices of the surface mesh. The novel approach which is also referred to “our improved approach” in what follows, uses the revised minima rule (RMR) to extract both concave and convex feature regions from the surface mesh of mechanical parts, and overcomes the inherent inadequacies of the original approach which is based on the minima rule (MR) and used for natural surface mesh models.

The remainder of the paper is organized as follows. The original approach based on MR is described in section 2, and the improved approach based on RMR is described in section 3. Section 4 outlines the experiments and a discussion through several cases, and the paper is concluded in section 5. It is noted here that in this paper the smaller of the two principal curvatures on the vertices of the surface triangular mesh is referred as the minimum curvature as defined in [16].

## Original Approach

One of the key theoretical bases of identifying feature regions from a surface mesh is derived from cognitive science, and the related theory is summarized in [17] and referred as the minima rule (MR): the human visual system is more likely to perceive regions of a surface that have negative minima of principal curvatures, which are shown as concave shape, compared with the coherency of different shaped parts on the surface model. In practice, these concave regions with negative minimum curvatures are often considered as interfaces separating shaped parts. Therefore, extracting the concave regions by the approach based on MR plays a significant role in the relevant research.

In [13], Lee et al. presented an original approach based on MR to extract feature regions, which would be described briefly below. Because the distribution of minimum curvatures on the vertices of a mesh may be compact or scattered for different surface mesh models, the ranges of the minimum curvatures would be too diverse in different models, therefore, it is not conducive to calculation, and thus, the minimum curvatures were normalized in the original approach. The normalized formula is expressed as Eq (1)

$$k_{N_{\text{min}}}(v)=(k_{\text{min}}(v)-\mu )/\sigma$$ (1)

where *k*_min_(*v*) is the minimum curvature on vertex *v*,

*μ* is the mean of *k*_min_(*v*) on all vertices of the surface mesh model,

*σ* is the standard deviation of *k*_min_(*v*) on all vertices of the surface mesh model, and

$k_{N_{\text{min}}}(v)$ is the normalized curvature on vertex *v*.

*σ* is deduced using Eq (2), where N is the number of vertices on the surface mesh model.

$$\sigma =\sqrt{\frac{1}{N}\sum_{i=1}^{N}{\left(k_{\text{min}}(v)-\mu \right)}^{2}}$$ (2)

Applying the normalized minimum curvature $k_{N_{\text{min}}}(v)$, a unique set of threshold parameters was utilized to extract feature regions over all targeted surface mesh models. Two thresholds of $k_{N_{\text{min}}}(v)$ act as parameters, namely, the upper bound is -0.8 and the lower bound is -1.2. Thus, those vertices, each of whose normalized minimum curvature $k_{N_{\text{min}}}(v)$ lies between -1.2 and -0.8, represent the desired concave feature regions on the surface mesh.

**Fig 1:**
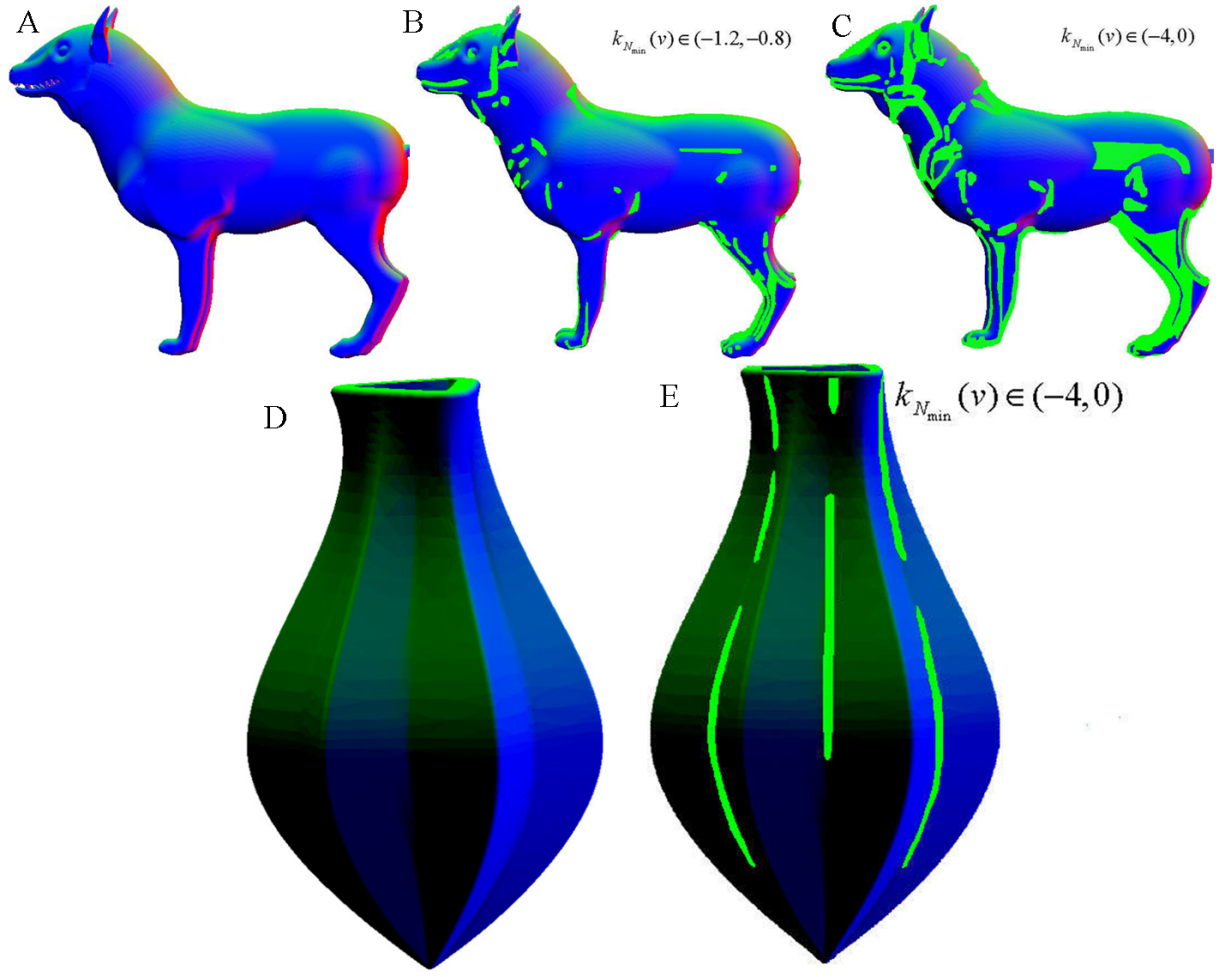
Illustration of the inadequacies of the original approach. (A) raw model of a wolf; (B) extracted feature regions of the wolf using a parameter range of (-1.2, -0.8); (C) extracted feature regions of the wolf using a parameter range of (-4, 0); (D) raw model of a juicer; (E) extracted feature regions of the juicer using a parameter range of (-4, 0).

However, after testing on a few mesh models, we found two inadequacies in the original approach for our objective in this paper as illustrated in Fig 1. Firstly, for reason that the original approach focus on salience of the feature regions, the range of selected normalized minimum curvature $k_{N_{\text{min}}}(v)$, which was uniform on all of the targeted models, was set to be narrow (-1.2,-0.8) to obtain narrow feature regions, but in this paper, we want to get into pieces of feature region to facilitate modeling the mechanical parts and so the range of selected normalized minimum curvature may be set to be more extensive such as the range (-4,0), whose results are illustrated in Fig 1A–1C. Secondly, the original approach is certainly effective to extract concave features with salience of cognitive science for natural models whose surface geometry smoothly and gradually varies and concave features are relatively important such as the wolf in Fig 1A–1C; however it is not especially effective to extract both concave and convex features for mechanical parts whose surface geometry drastically varies and where concave and convex features are equally important when modeling, e.g., a juicer as shown in Fig 1D and 1E. Note that in all of the extracted result figures in this paper, the concave features are rendered in green on the surface mesh models, while the convex features are in red.

**Fig 2:**
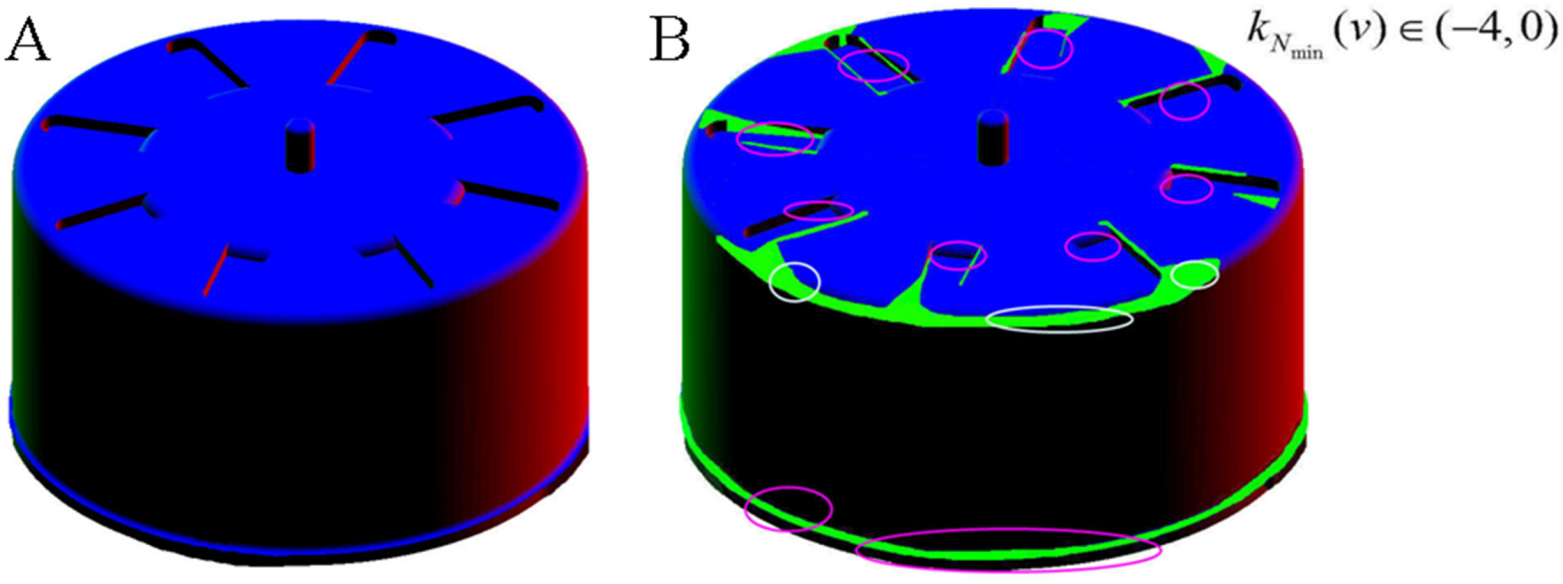
Illustration of the theoretical inadequacy of the original approach for our objective in the paper. (A) raw model of a gear shell; (B) extracted feature regions of the gear shell using a parameter range of (-4, 0).

Furthermore, the original approach has certain theoretical inadequacy for the objective of this paper. Normalization of the minimum curvatures in the original approach is not fully theoretically justified when applied to the mechanical parts whose surface geometry drastically varies and where concave and convex features are equally important when modeling, but this inadequacy doesn’t perform effectively on the natural models for reason of their surface geometry smoothly and gradually varies and concave features are relatively important. Because the expectation *μ* of the minimum curvatures on all vertices each surface mesh model is not always zero, the relationship between the surface mesh shape and the two measures of the minimum curvature (the expectation *μ* and standard deviation *σ*) becomes uncertain. For reason of this uncertainty, a distortion of the geometrical sense of the natural curvature appears, and thus, the following consequence might be resulted in: for these surface mesh models with mostly flat or convex area, a few flat or convex portions might be incorrectly identified as concave features, i.e., ineffective or error feature regions are produced, as illustrated in Fig 2A and 2B where only a portion of concave features were extracted(such as the lower edge concave feature denoted by pink circle), some other concave features (such as the grooves on the upper flat surface denoted by pink circles) could not be resulted in, and a few convex or flat features were mistakenly extracted (such as the upper edge convex features and its neighboring area denoted by white circles).

## Improved Approach Based on RMR

The original approach based on MR described in section 2 could only extract concave feature regions from natural surface mesh models. However, the resulting concave features are far from availablity in practical research and application. In reverse engineering, sharp features containing convex and concave shapes are important hints for modeling; there are a variety of graphic applications where convex regions are equally important as concave regions. Thus, this paper considers the simultaneous extraction of both concave and convex feature regions from the surface mesh model.

MR could only be utilized to extract concave features from the natural surface model. To further obtain convex features for the objective of this paper, **the revised minima rule (RMR)** is introduced. As expressed in (Formula 3), surface mesh areas that possess vertices each of whose minimum curvature is less than or greater than the corresponding preset threshold (*τ*_*d*_ or *τ*_*u*_), are defined as feature regions. Naturally the defined feature regions include both concave and convex features. As the improved approach of mesh segmentation in this paper, we use RMR to extract concave and convex feature regions from the surface mesh model of mechanical parts. In what follows, we would progressively expand the narrative on the improved approach.

$$\left\{v_{fea}|k_{\text{min}}(v)\in (-\infty ,\tau_{d})\cup (\tau_{u},+\infty ),v\in mesh\right\}$$ (3)

In (Formula 3), *v*_*fea*_ are the required feature vertices, *τ*_*d*_ is the limit of the lower range, and *τ*_*u*_ is the limit of the upper range.

### 3.1 Our approach to extract feature vertices

To overcome the inherent inadequacies of the original approach for our objective, i.e., to maintain the nature of the geometric sense of the original curvature distribution on the surface mesh, and to further identify convex features as an extension of the original approach, we develop an improved approach based on RMR, which uses the natural logarithmic function of the minimum curvatures on the vertices of the surface mesh.

The idea of defining the features in terms of the logarithm of the curvature has arisen before [14]. However, this previous idea is different from ours, whose function was the logarithm of a probability density function (which is solved based on principle of cognitive science) with regard to curvature, and its objective was same as the original approach to extract concave features. While the objective of our improved approach is to extract both concave and convex features, we adopt the logarithmic function with regard to the minimum curvature to meet the requirement, and there is no subjective factor of human being involved in our improved approach instead of that in [14].

In this paper, the reason for the choice of the natural logarithmic function with regard to the minimum curvature rather than some other function was based on the following two analytical terms.

**Fig 3:**
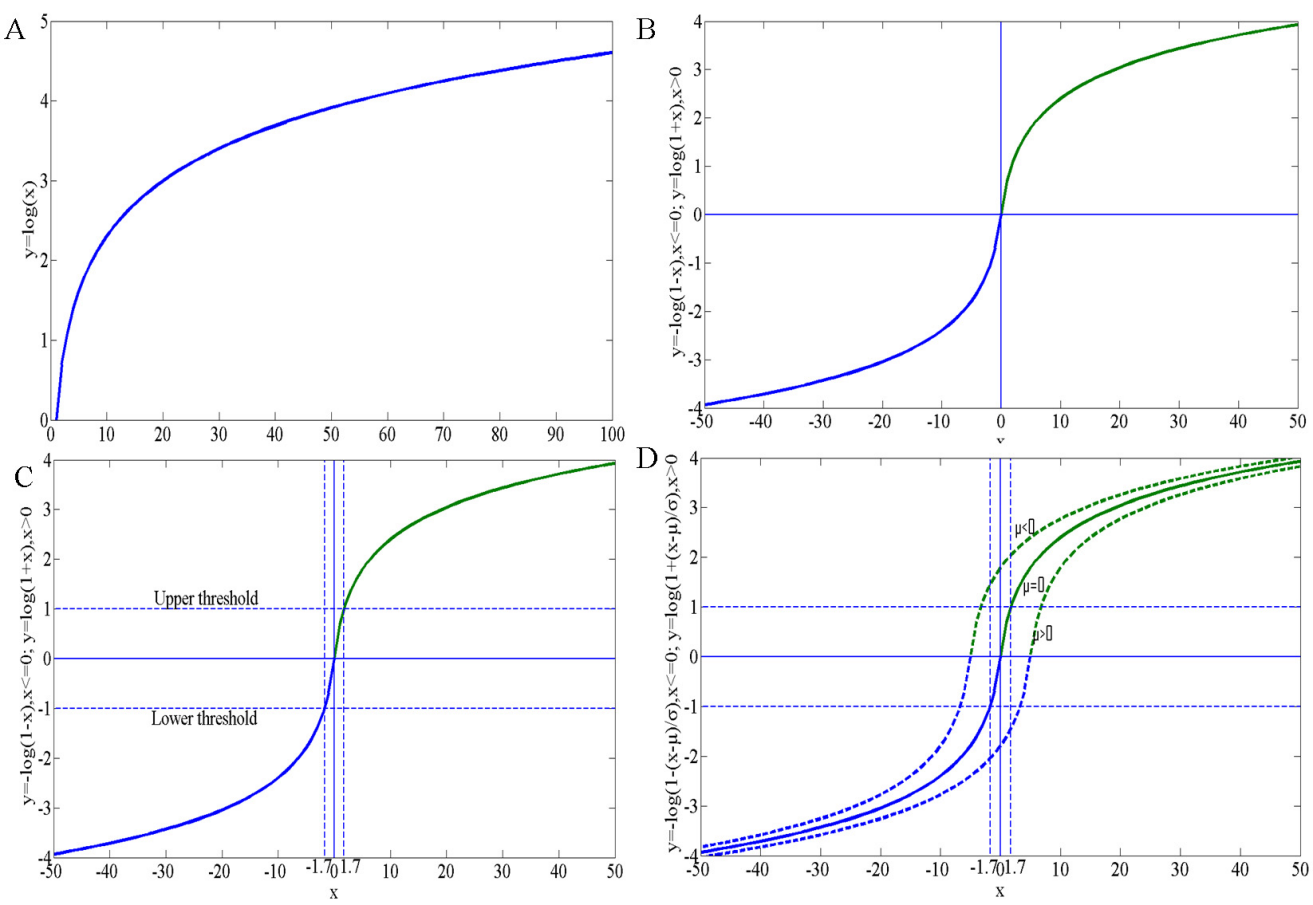
Illustration of the chosen logarithmic function. (A) natural logarithmic function; (B) logarithmic function of (Formula 4); (C) range of the selected thresholds shown by the dashed lines; (D) distortion shown by the dashed curves when normalized by the expectation *μ*

1. For obtaining the feature vertices according to RMR, the key principle of choosing the function is to enable the easy selection of the threshold parameters which are the dependent variables of the function. Because the ranges of the minimum curvatures on different surface models may be extensive even to (−∞,+∞), it is necessary for the chosen function to narrow the value range of the minimum curvatures to be convenient to select the thresholds. As illustrated in Fig 3A, the logarithmic function narrows the range (0,100)of *x* into (0,5) of *y* and may perform the compression more rapidly as the independent variable increases.
2. Referring to (Formula 3), to make the user select the thresholds objectively according to the natural minimum curvatures among some range from which to select the thresholds *τ*_*d*_ and *τ*_*u*_, each of the chosen function values should only be slightly different from the corresponding independent variables namely the minimum curvatures. In other words, the chosen function should be close to the linear function *y* = *x* in the range of the independent variable from which the thresholds are selected from.

Based on the above considerations, the natural logarithmic function expressed as (Formula 4) (referring Fig 3B) was chosen to address the minimum curvatures. Specifically, the improved approach is formulated as Eqs (4) and (5):

$$k_{Log_{\text{min}}}(v)=\{\begin{array}{ll} \text{log}(1+k_{\text{min}}(v)), & k_{\text{min}}(v)\geq 0\\ -\text{log}(1-k_{\text{min}}(v)), & k_{\text{min}}(v)<0 \end{array}$$ (4)

$$\left\{v_{fea}|k_{Log_{\text{min}}}(v)\in (-\infty ,\tau_{Ld})\cup (\tau_{Lu},+\infty ),v\in mesh\right\}$$ (5)

In (Formula 5), *v*_*fea*_ are the required feature vertices, *τ*_*Ld*_ is the limit of the lower range, and *τ*_*Lu*_ is the limit of the upper range.

We have verified that (Formula 4) corresponds to the analytical term (II) as follows: the second order Taylor expansion of (Formula 4) is (Formula 6).

$$\{\begin{array}{ll} \text{y}=\text{log}(1+x)=x-x^{2}+\frac{f^{\left(3\right)}(\theta (x))}{3!}x^{3} & x\geq 0\\ \text{y}=-\text{log}(1-x)=x+x^{2}+\frac{f^{\left(3\right)}(\theta (x))}{3!}x^{3} & x<0 \end{array}$$ (6)

Referring to Fig 3B, in the Taylor expansion Eq (6), higher-order terms of the argument *x* (e.g. *x* ^2^ and x^3^) in the range near the origin (e.g. (−1,1)) might be omitted due to their small values; thus, Formulas (4) or (6) approximates the linear function *y* = *x* in the range near the origin which maps the geometric sense that the natural minimum curvature (represented by the argument *x* in Fig 3) expresses.

However, the differences between $k_{Log_{\text{min}}}(v)$ of neighboring vertices are too diverse for various surface mesh models, and thus setting thresholds *τ*_*Ld*_ and *τ*_*Lu*_ in (Formula 5) remains a difficult task for different models. To solve this problem, we attempt to produce a uniform range from which the thresholds could be selected; thus, the standard deviation *σ* of *k*_min_(*v*) on all vertices of the surface mesh model is introduced and so (Formula 7) is created.

$$k_{Log_{\text{min}}}(v)=\{\begin{array}{ll} \text{log}(1+k_{\text{min}}(v)/\sigma ), & k_{\text{min}}(v)\geq 0\\ -\text{log}(1-k_{\text{min}}(v)/\sigma ), & k_{\text{min}}(v)<0 \end{array}$$ (7)

By favor of (Formula 7), the thresholds *τ*_*Ld*_ and *τ*_*Lu*_ in (Formula 5) may be selected from a small value range to obtain the features. Through our tests for most of the surface mesh models, the feature vertices whose $k_{Log_{\text{min}}}(v)\in (-\infty ,-1)\cup (1,+\infty )$ account for a small proportion of all of the desired features on a surface mesh model. Thus (−1,0) and (0,1) are the appropriate range for *τ*_*Ld*_ and *τ*_*Lu*_, respectively, to be selected from for all the targeted surface mesh models to obtain each of whose desired features, which are shown by the dashed lines in Fig 3C. Moreover, expectation *μ* in the improved approach should be reconsidered as shown in (Formula 8)(which is the final determinant of $k_{Log_{\text{min}}}(v)$) because distortion would be resulted in due to *μ* ≠ 0 as shown by the dashed curves in Fig 3D if not be considered.

$$k_{Log_{\text{min}}}(v)=\{\begin{array}{ll} \text{log}(1+(k_{\text{min}}(v)-\mu )/\sigma ), & k_{\text{min}}(v)\geq 0\\ -\text{log}(1-(k_{\text{min}}(v)-\mu )/\sigma ), & k_{\text{min}}(v)<0 \end{array}$$ (8)

In practice, it is difficult to determine the specific number of feature elements for a surface mesh model because it greatly differs on various models. Given the complexity of human intervention, we developed an automated program to obtain the desired feature vertices without setting the thresholds *τ*_*Ld*_ and *τ*_*Lu*_ according to Formulas (8) and (5). It is as follows and assumes that, ideally feature vertices account for 10% of the total number of surface mesh vertices. The experiments demonstrated that this assumption is reliable.

1. Set the initial thresholds as $\{\begin{array}{l} \tau_{Ld}=-1\\ \tau_{Lu}=1 \end{array}$; then extract the feature vertices according to Formulas (5) and (8). If the accounted proportion of extracted feature vertices is greater than the range of 9–11% of the total number of surface mesh vertices, return; else, reset the thresholds as $\{\begin{array}{l} \tau_{Ld}=-0.5\\ \tau_{Lu}=0.5 \end{array}$, *a* = 0.5, to perform the following steps.
2. For the current thresholds $\{\begin{array}{l} \tau_{Ld}=-a\\ \tau_{Lu}=a \end{array}$, *a*∈(0,1), extract the feature vertices according to Formulas (5) and (8).
3. If the accounted portion of the extracted feature vertices is greater than 11% of the total number of surface mesh vertices, reset the thresholds as $\{\begin{array}{l} \tau_{Ld}=-a-0.5*a\\ \tau_{Lu}=a+0.5*a \end{array}$; then execute the re-assignment *a* = *a*+0.5*a, and perform step (2) with this re-assignment.
4. If the accounted portion of extracted feature vertices is smaller than 9% of the total number of surface mesh vertices, reset the thresholds as $\{\begin{array}{l} \tau_{Ld}=-a+0.5*a\\ \tau_{Lu}=a-0.5*a \end{array}$; then execute the re-assignment *a* = *a*−0.5*a, and perform step (2) with this re-assignment.
5. Perform steps (2), (3) and (4) iteratively until the proportion of feature vertices is as far as possible between 9–11% of the total number of the surface mesh vertices.

### 3.2 Search for the feature edges and facets

Above is the part of our improved approach to obtain the feature vertices, whereas the basic elements constituting the surface mesh model are triangular facets that most mesh processing operations target on, therefore it is necessary to convert these feature vertices into regions of feature edges and further into regions of feature facets.

**Fig 4:**
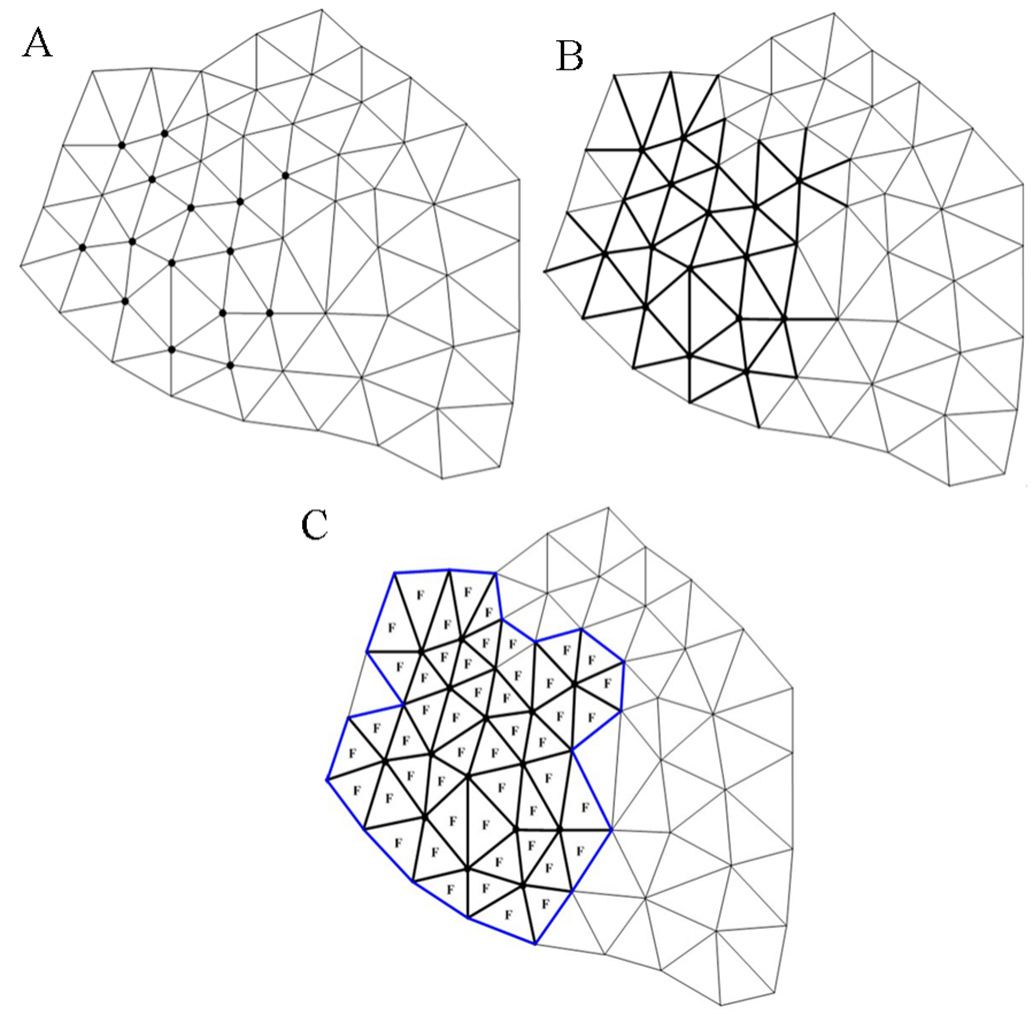
Illustration of searching for the feature edges and facets. (A) feature vertices are shown in bold in the surface mesh; (B) feature edges are shown in bold; (C) feature facets are marked as “**F**”, and the boundary of the feature region is shown in bold blue.

The rule of searching for the feature edges and facets is simple as illustrated in Fig 4A–4C, i.e., an edge that possesses at least one feature vertex is defined as a feature edge and a triangular facet that possesses at least one feature edge is defined as a feature facet.

## Experiments and Discussion

The original approach, our improved one and some other latest conventional approaches have been implemented in VC++6.0 and OpenGL. The experiments were performed on a Windows8 PC with 2.60 GHz Intel Celeron CPU and 2.0 GB of RAM.

**Fig 5:**
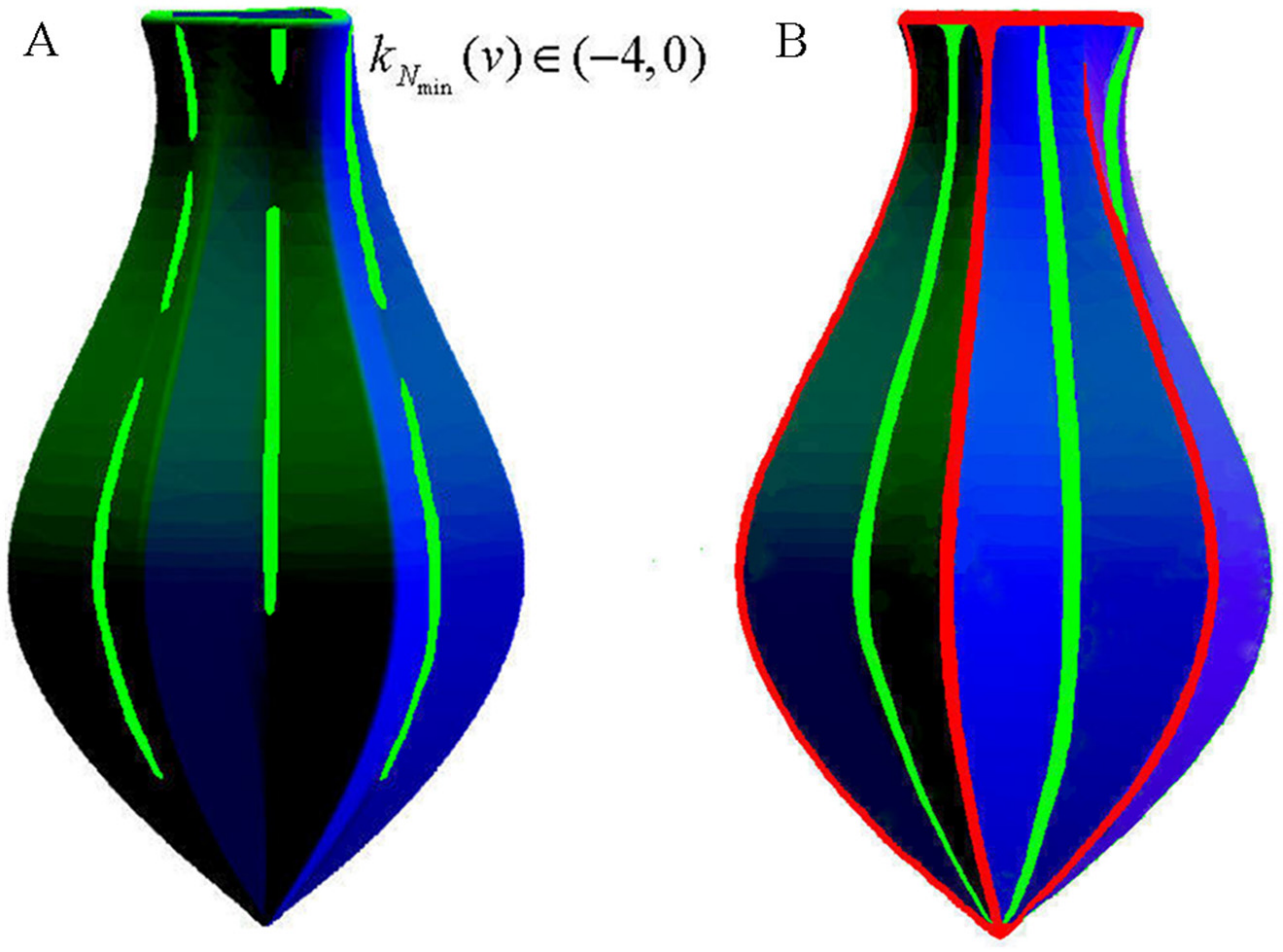
The experiments on the juicer model as mechanical part. (A) result by the original approach; (B) result by our improved approach.

Our improved approach has inherited the functionality of the original approach to extract concave features, and as an extension it could identify the concave and convex features simultaneously for mechanical parts (whose surface geometry dramatically varies and where concave and convex features are equally important), whereas this effect could not be achieved by the original approach. In all the experimental results, the concave features are rendered in green and the convex features are in red. The comparison of experiments on the juicer model as mechanical part is shown in Fig 5A and 5B, and the better results obtained by the improved approach could be clearly observed in Fig 5B. As shown in Fig 5A, concave features could only be extracted on the concave ridges and no features could be identified on the convex ridges, besides the extracted concave features are too fragmented when the original approach acts on mechanical parts whose surface geometry varies drastically. In contrast as shown in Fig 5B, our improved approach could extract the concave and convex features perfectly on juicer model as mechanical part, and the extracted regions are coherent and not fragmented. Therefore our improved approach is more suitable to extract feature regions than the original approach for the mechanical parts whose surface geometry varies drastically and where concave and convex features are equally important when modeling.

**Fig 6:**
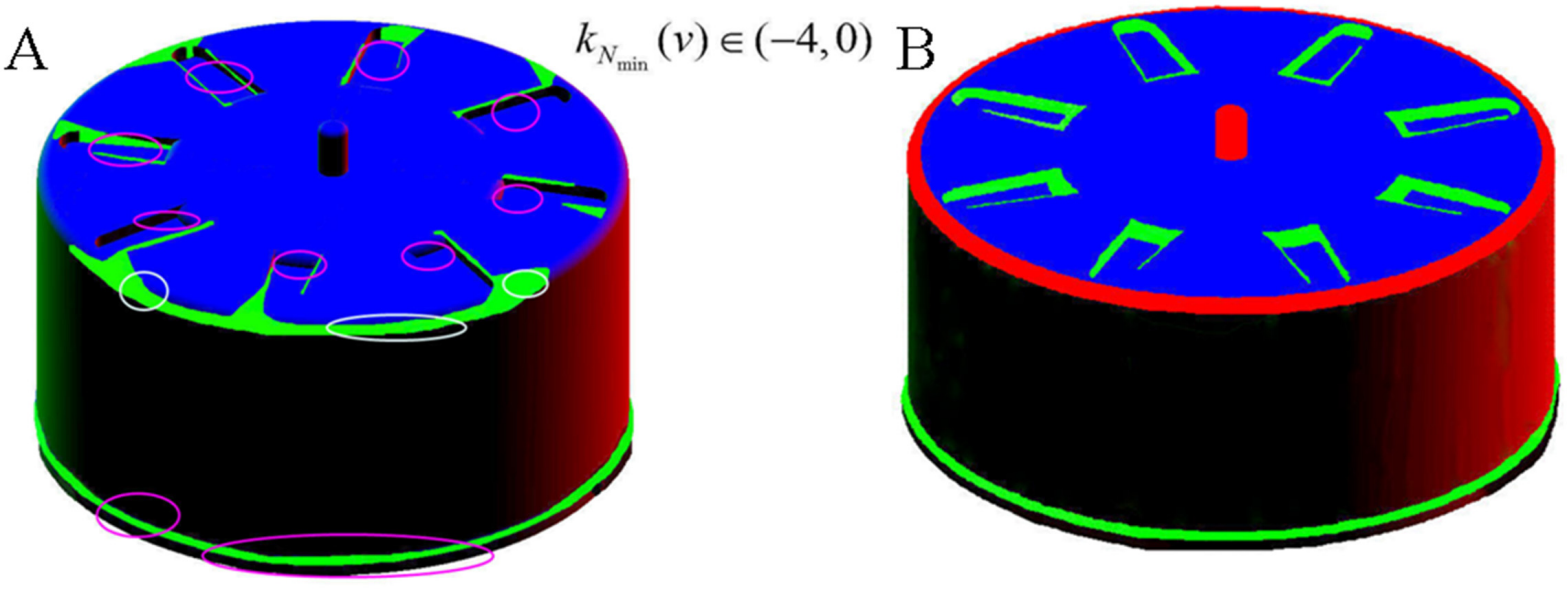
Experiments on the gear shell as a mechanical part whose flat or convex regions are dominant. (A) result by the original approach; (B) result by the improved approach.

As described above, in the original approach, a distortion of the geometrical sense of the natural curvature arises because of normalization of the minimum curvatures; thus, poor results (as shown in Fig 6A) might appear when the targeted surface mesh model is dominant by flat or convex regions. However the improved approach avoids this inadequacy and obtains a perfect effect, as demonstrated by the comparison of experiments on the gear shell as shown in Fig 6A and 6B. The sound result shown in Fig 6B, containing almost all of the concave and convex features, were obtained by our improved approach which is far better than what’s done by the original approach as shown in Fig 6A. Furthermore, the improved approach realizes automation without setting parameters and thus avoids the influence of human intervention.

**Fig 7:**
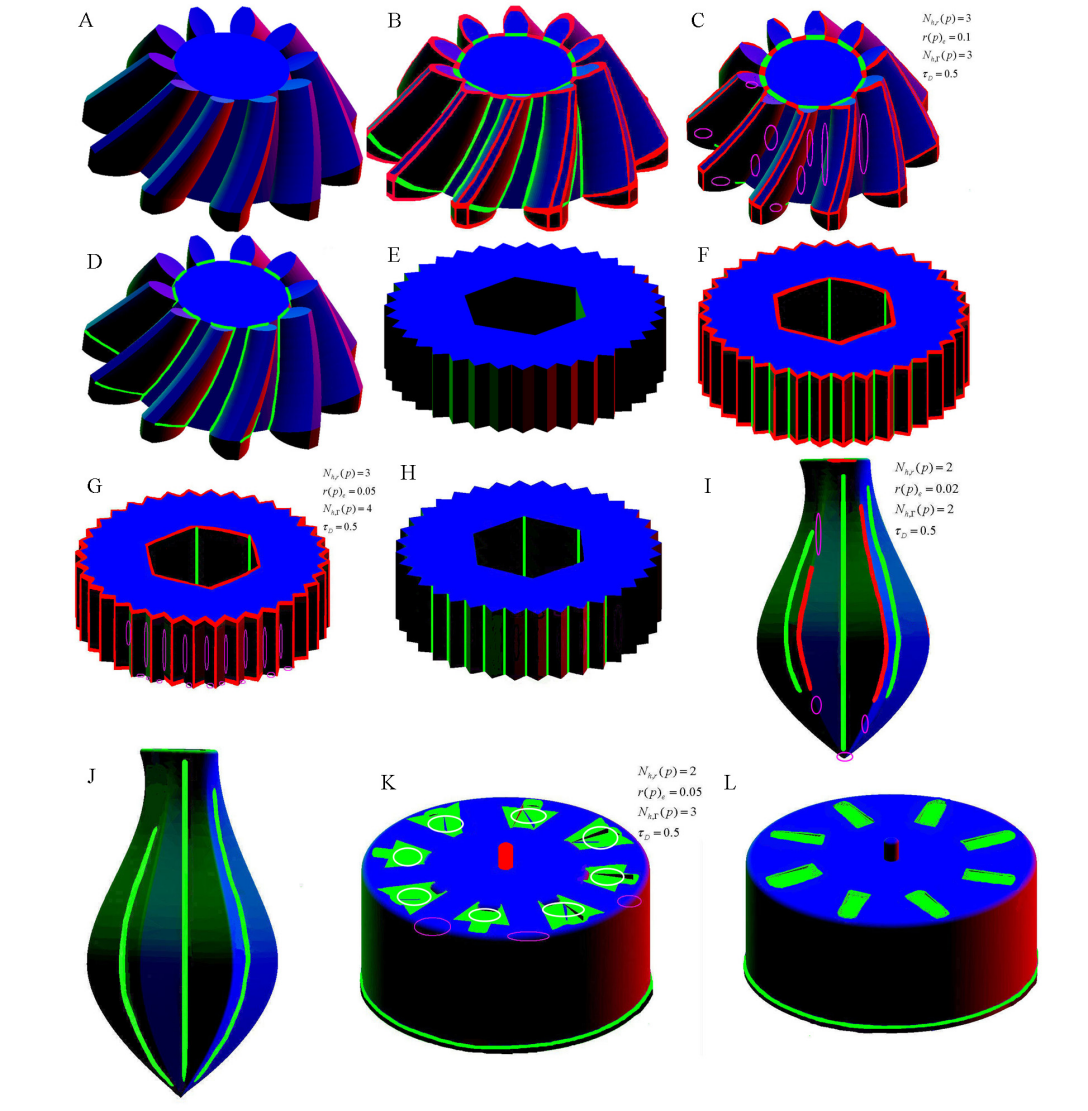
Experiments on comparison of our improved method with two newest conventional approaches. (A) raw model of a bevel gear; (B) result by the improved approach on the bevel gear; (C) result by the approach in [15] on the bevel gear; (D) result by the approach in [14] on the bevel gear;(E) raw model of a screw bit; (F) result by the improved approach on the screw bit; (G) result by the approach in [15] on the screw bit; (H) result by the approach in [14] on the screw bit; (I) result by the approach in [15] on juicer; (J) result by the approach in [14] on juicer; (K) result by the approach in [15] on the gear shell; (L) result by the approach in [14] on the gear shell.

In addition to the above characteristics of the improved approach compared to the original approach, the improved approach performs better, due to its more appropriate principle which is based on processed minimum curvatures, than the newest related representative approaches in [14, 15]. Here, two cases of a bevel gear and a screw bit along with the juicer and the gear shell are used in our experiments to illustrate the superiority of our improved approach via comparing with the two newest representative approaches based on the fitting-error and cognitive science in [15] and [14] respectively. Fig 7B and 7F show that feature regions have been comprehensively identified by the improved approach respectively on the models of bevel gear and screw bit; while a few features might not be identified which are marked by pink circles by the approach in [15] as shown in Fig 7C and 7G, and convex features could not be extracted by the approach in [14] as shown in Fig 7D and 7H. As for the two other models: the juicer and the gear shell, the experiments by the approaches in [15,14] are illustrated in Fig 7I, 7J, 7K and 7L respectively. In Fig 7I and 7J, many significant concave and convex features of the juicer have not been identified (such as the places denoted by pink circles in Fig 7I). In Fig 7K, the upper boundary portion of the cylindrical surface (denoted by pink circles) of the gear shell was not identified as a convex feature by the approach in [15]; in addition denoted by white circles, confusion appears when distinguishing the grooves on the upper flat surface where surface geometry varies dramatically. In Fig 7L, only the concave features were identified, and the convex ones were ignored because of the inherent principle of the approach in [14]; furthermore, the extracted concave features including the flat bottom of the grooves are different from what has been obtained by our improved approach, and thus it might be considered that the approach in [14] lacks accuracy compared with our approach when dealing with mechanical parts with dramatic geometry. In summary, based on the above discussion, our improved approach could correctly identify both concave and convex feature regions, while the other two representative conventional approaches sometimes produce errors or could not identify the corresponding features on certain locations.

**Fig 8:**
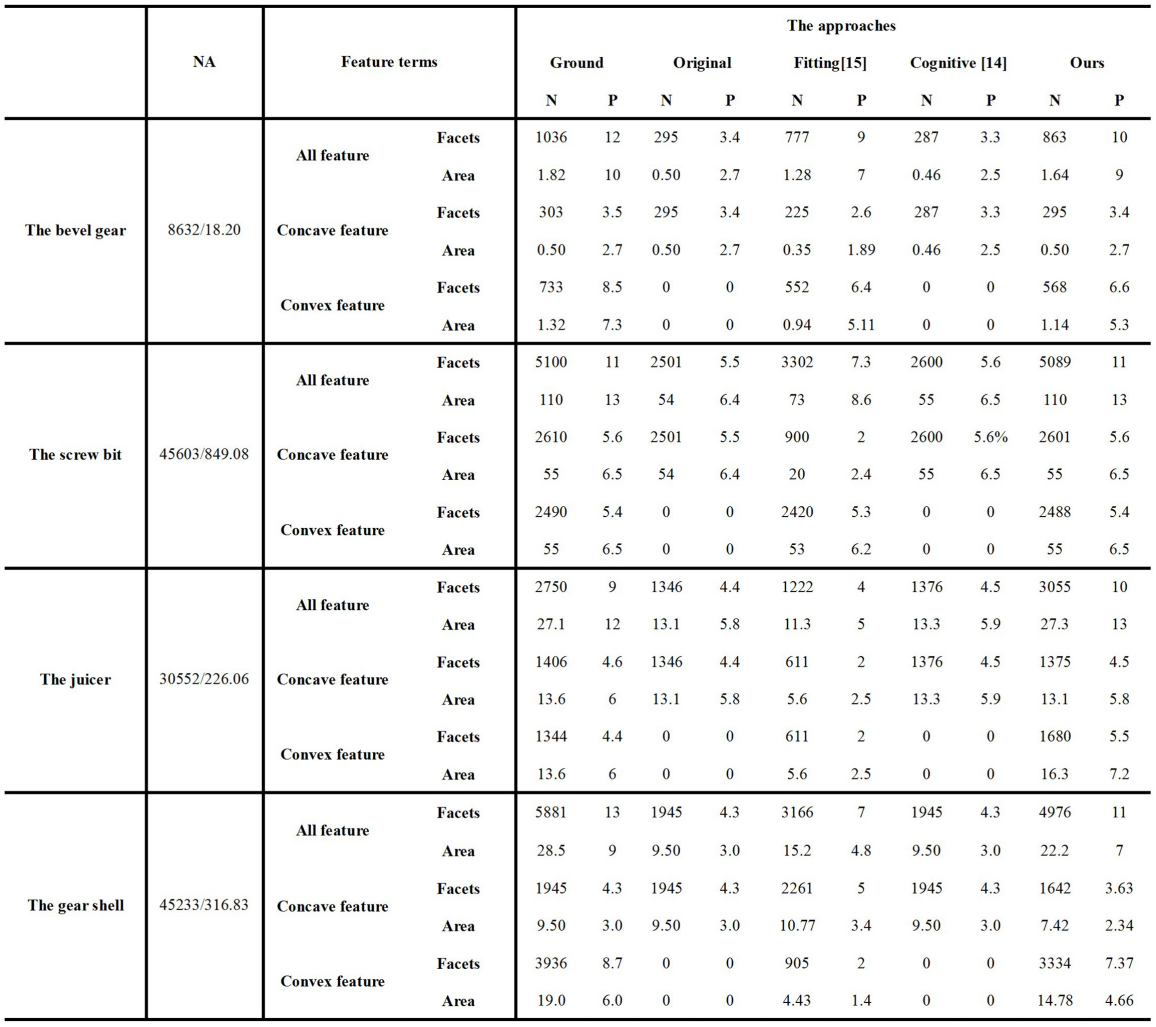
A quantitative comparison of experiments by the approaches.

Furthermore, quantitative evaluation of the improved approach could be performed by comparing, in terms of the number of feature components and their areas, with the manually created ground-truth approach, the original approach, the fitting-error approach in [15] and the cognitive approach in [14]. Among them, the manually created ground-truth approach is to segment the mesh model manually, whose results may be considered as the actual concave features and convex features on the targeted model. A quantitative comparison of experiments by the above several approaches is illustrated in Fig 8.

In Fig 8, NA represents the number of the feature facets or the value of feature area, P represents proportion of the feature facets/area accounted for all of the facets/area of the model (whose unit is “%”); NA represents “Number of facets/area of surface (mm^2^)”; "Ground" represents Ground-truth approach; "Original" represents Original approach; "Fitting[15]" represents Fitting-error approach in [15]; "Cognitive[14]" represents Cognitive approach in [14]; “Ours” represents Our improved approach.

Fig 8 clearly indicates that the extracted feature regions obtained by our improved approach are the closest to the ground-truth features among the four compared approaches. Referring to the data of the gear shell in Fig 8, although concave features extracted by the original approach and the cognitive approach in [14] are better than those extracted by our improved approach, these two cognitive approaches could only identify concave features due to the limitation of their principle, and so they are not applicable for mechanical parts in modeling. In addition referring the corresponding data of our improved approach and the fitting-error approach in [15], our improved approach, which assures the correctness of the results referring the experimental Figures, could extract more feature regions compared with the fitting-error approach in [15], and so the results of our improved approach are more close to the actual results. Briefly, our improved approach is superior to the other newest conventional approaches, as demonstrated by the data in Fig 8.

Based on the above experiments, it could be concluded that

1. The improved approach is superior to the original approach when applied on those mechanical part surface models especially where flat and convex features are dominant, in which case the original approach makes false judgments on normal features.
2. The improved approach could extract both concave and convex feature regions from the surface mesh of mechanical parts and provides an improved performance compared to the newest conventional approaches

## Conclusion

The objective of this paper is to extract both concave and convex feature regions from a surface mesh model of mechanical part whose surface geometry dramatically varies and where concave and convex features are equally important when modeling. Although the original approach based on MR motivated by cognitive science is a most convenient way to extract concave feature regions, it is only effective for natural surface mesh models. To extend the advantages of the original approach and consider convex features, we propose the improved approach based on the developed RMR. The improved approach could be performed automatically without setting parameters, could extract simultaneously concave and convex feature regions more accurately and overcomes the inherent inadequacies of the original approach when applied to mechanical parts in modeling. Furthermore, our approach has been demonstrated to be superior to the newest conventional approaches through experimental tests. As for the future, our improved approach based on RMR may provide important reference significance for geometric segmentation and feature extraction in practice.
